# Diet disparity among sympatric herbivorous cichlids in the same ecomorphs in Lake Tanganyika: amplicon pyrosequences on algal farms and stomach contents

**DOI:** 10.1186/s12915-014-0090-4

**Published:** 2014-10-29

**Authors:** Hiroki Hata, Akifumi S Tanabe, Satoshi Yamamoto, Hirokazu Toju, Masanori Kohda, Michio Hori

**Affiliations:** Graduate School of Science and Engineering, Ehime University, 2-5 Bunkyo, Matsuyama, Ehime Japan; Graduate School of Global Environmental Studies, Kyoto University, Sakyo, Kyoto Japan; National Research Institute of Fisheries Science, Fisheries Research Agency, 2-12-4 Fukuura, Kanazawa, Yokohama Japan; Graduate School of Human and Environmental Studies, Yoshida-Nihonmatsu, Kyoto University, Sakyo, Kyoto Japan; Graduate School of Science, Osaka City University, Sumiyoshi-ku, Osaka Japan; Kyoto University, Yoshida-Honmachi, Sakyo, Kyoto Japan

**Keywords:** Adaptive radiation, Tanganyikan cichlid, Herbivore

## Abstract

**Background:**

Lake Tanganyika, an ancient lake in the Great Rift Valley, is famous for the adaptive radiation of cichlids. Five tribes of the Cichlidae family have acquired herbivory, with five ecomorphs: grazers, browsers, scrapers, biters and scoopers. Sixteen species of the herbivorous cichlids coexist on a rocky littoral slope in the lake. Seven of them individually defend feeding territories against intruding herbivores to establish algal farms. We collected epiphyton from these territories at various depths and also gathered fish specimens. Algal and cyanobacteria community structures were analysed using the amplicon-metagenomic method.

**Results:**

Based on 454-pyrosequencing of SSU rRNA gene sequences, we identified 300 phototrophic taxa, including 197 cyanobacteria, 57 bacillariophytes, and 31 chlorophytes. Algal farms differed significantly in their composition among cichlid species, even in the same ecomorph, due in part to their habitat-depth segregation. The algal species composition of the stomach contents and algal farms of each species differed, suggesting that cichlids selectively harvest their farms. The stomach contents were highly diverse, even between species in the same tribe, in the same feeding ecomorph.

**Conclusions:**

In this study, the amplicon-metagenomic approach revealed food niche separation based on habitat-depth segregation among coexisting herbivorous cichlids in the same ecomorphs in Lake Tanganyika.

**Electronic supplementary material:**

The online version of this article (doi:10.1186/s12915-014-0090-4) contains supplementary material, which is available to authorized users.

## Background

The cichlid species flock in Lake Tanganyika is a model system of adaptive radiation, defined as the rapid evolution of a multitude of species from a common ancestor as a consequence of their adaptation to various ecological niches. After the formation of the lake, 9 to 12 million years ago, several ancestral strains diversified into more than 200 species in 14 tribes [1-6]. Tanganyikan cichlids are unique because of the species richness of each of the ecomorphs [7,8] that have evolved convergently and coexist in similar habitats, not only among different lakes but also within Lake Tanganyika [4]. Additionally, only six of the fourteen tribes present in Lake Tanganyika show sexual dichromatism; the others are monochromatic lineages [5,9], in contrast to other African lakes in which the degree of cichlids’ adaptive radiation correlates highly with the presence of sexual dichromatism [5].

In Lake Tanganyika, five tribes of the family Cichlidae (Tropheini, Lamprologini, Ectodini, Eretmodini, and Tilapini) have acquired several ecomorphs that are closely related to feeding habits in herbivory such as grazing, browsing, scraping, biting, and scooping [10-13]. These tribes have no sexual dichromatism, and therefore, we can focus on the effect of ecological opportunity in the adaptive radiation of these herbivorous cichlids [14,15]. Grazers, browsers, and scrapers are highly specialised and diversified, especially in these herbivorous habits [16]. Grazers comb unicellular algae from epilithic assemblages using multiple rows of similar-sized slender teeth with fork-like tricuspid tips [17,18]. Browsers nip and nibble filamentous algae using their bicuspid teeth, which line the outermost of both jaws [11,19]. Scrapers rub epiphyton from rock surfaces using several rows of chisel-like teeth [20]. The fishes of each ecomorph have distinct specialised trophic morphologies in their jaw structures and intestine lengths [21,22], physiological abilities, such as secretion of digestive enzymes [21] and behaviours, such as cropping frequency [19].

In one rocky littoral area of the lake, 16 species of herbivorous cichlids coexist [23]. Seven of them individually defend their feeding territories against intruding herbivores to establish algal farms on which the territory-holder feeds [24-29]. Grazers and browsers utilise species-specific depth ranges, and species in the same depth range choose the substratum types on which they feed [10,23,30]. In particular, territorial cichlids of the same ecomorph exhibit separate habitat depths [29,31,32], and ecological character displacement in habitat depth and associated body shape were reported in sympatric populations of *Tropheus moorii* and *Tropheus polli*, compared to isolated populations of the two species [33].

Niche segregation leading to multi-species coexistence has at least three axes: space, food and time [34]. These herbivorous cichlids are diurnally active and do not have separate active times during the day [35]. In this study, we focused on food and habitat depth. How do feeding specialisation and habitat-depth selection cause food-source segregation, thereby enabling the coexistence of multiple species in the same ecomorph in this adaptive radiation system? To answer this question, the degree of diet disparity in algae and cyanobacteria between herbivorous cichlid species was analysed at the species-level using a metagenomic amplicon sequencing approach. Based on 454-pyrosequencing of SSU rRNA gene sequences, we described algal and cyanobacterial compositions in algal farms and the stomach contents of herbivorous cichlid species on a rocky slope of Lake Tanganyika. The composition of algal farms was compared among ecomorphs and among species within each ecomorph, and the effect of habitat depth on the composition was analysed. The stomach contents were compared among ecomorphs and among species within each ecomorph, and were compared with algal farm composition. Finally, the relationships between the phylogenetic distances between fish species and the similarity of algal farms and stomach contents were analysed.

## Results

We sampled 16 species of herbivorous cichlids (*Oreochromis tanganicae*, *Xenotilapia papilio*, *Eretmodus cyanostictus*, *Telmatochromis temporalis*, *Telmatochromis vittatus*, *Variabilichromis moorii*, *Interochromis loocki*, *Pseudosimochromis curvifrons*, *Petrochromis famula*, *Petrochromis fasciolatus*, *Petrochromis macrognathus*, *Petrochromis polyodon*, *Petrochromis horii*, *Petrochromis trewavasae*, *Simochromis diagramma*, and *Tropheus moorii*) from Kasenga Point (8°43′ S, 31°08′ E) near Mpulungu, Zambia, on the southern tip of Lake Tanganyika in November 2010 (Table 1). Periphyton samples were simultaneously collected from five territories each of *T. temporalis*, *V. moorii*, *P. macrognathus*, *P. polyodon*, *P. horii*, *P. trewavasae*, *T. moorii*, dominant males of *I. loocki* and *P. curvifrons* (in these species females and inferior males do not have feeding territory, Table 1), and breeding pairs of *E. cyanostictus* and *X. papilio.* Another 13 periphyton samples were collected from outside the cichlid territories. Total DNA was extracted from each of the stomach content samples of the cichlids and periphyton samples, and pyrosequencing of SSU rRNA gene was conducted. The resulting 5,073 consensus sequences represented operational taxonomic units (OTUs; Additional file 1: Data S1). Of the 5,073 consensus reads, 2,663 reads were excluded as singletons because such sequences are putatively erroneous. Taxonomic assignment of the OTUs was performed by the Query-centric auto-k-nearest-neighbour (QCauto) method, using the software Claident v. 0.1.2012.05.21 [36,37].

**Table 1:** **Herbivorous cichlids in Lake Tanganyika and their ecomorphs, territoriality and the number of samples**

**Tribe**	**Species**	**Abbreviation**	**Feeding ecomorph**	**Feeding territory**	**Number of algal farms**	**Sampling depth (m)**	**Number of stomach contents**	**References**
Tilapiini	*Oreochromis tanganicae*	Otan	biter	no	-		4(1)	[4,38,39]
Ectodini	*Xenotilapia papilio*	Xpap	scooper	breeding pairs only	5 (4)	7.9 (5.3 to 11.4)	4(3)	[39,40]
Eretmodini	*Eretmodus cyanostictus*	Ecya	scraper	breeding pairs only	5 (5)	2.2 (1.9 to 2.4)	6(2)	[41,42]
Lamprologini	*Telmatochromis temporalis*	Ttem	browser	yes	5 (3)	8.1 (2.4 to 19.6)	5(1)	[10,43]
Lamprologini	*Telmatochromis vittatus*	Tvit	browser	no	-		5(3)	[44]
Lamprologini	*Variabilichromis moorii*	Vmoo	browser	yes	5 (5)	4.6 (2.5 to 6.7)	6(4)	[45]
Tropheini	*Interochromis loocki*	Iloo	grazer	dominant males only	5 (5)	6.8 (3.1 to 13.0)	5(1)	[46]
Tropheini	*Pseudosimochromis curvifrons*	Pcur	browser	dominant males only	5 (5)	1.3 (1.0 to 2.1)	3(2)	[10,24]
Tropheini	*Petrochromis famula*	Pfam	grazer	dominant males only	-		1(0)	[26]
Tropheini	*Petrochromis fasciolatus*	Pfas	grazer	dominant males only	-		5(3)	[26-28]
Tropheini	*Petrochromis macrognathus*	Pmac	grazer	yes	5 (5)	0.3 (0.3 to 0.4)	4(1)	[30]
Tropheini	*Petrochromis polyodon*	Ppol	grazer	yes	5 (5)	3.0 (2.5 to 3.3)	5(1)	[10]
Tropheini	*Petrochromis horii*	Phor	grazer	yes	5 (4)	15.2 (15.0 to 15.7)	5(2)	[47]
Tropheini	*Petrochromis trewavasae*	Ptre	grazer	yes	5 (5)	10.1 (6.4 to 13.7)	5(3)	[10]
Tropheini	*Simochromis diagramma*	Sdia	browser	no	-		5(0)	[10]
Tropheini	*Tropheus moorii*	Tmoo	browser	yes	5 (5)	8.7 (6.0 to 10.5)	5(1)	[10,19,25]
-	Algal assemblages outside cichlid territories	Out	-	-	13 (9)	13.7 (3.2 to 27.5)	-	

Numbers in parentheses are the numbers of samples with more than 40 reads of phototrophic OTUs, which were used in subsequent analyses. Sampling depths indicate the depths at which algal farm samples were collected, shown as averages (minimum-maximum). OTUs, operational taxonomic units.

In total, we identified 300 phototrophic OTUs (see Additional file 1: Data S1). The algal farms of cichlids, periphytons outside the territories and stomach contents comprised 31.6 ± 12.2 phototrophic OTUs (average ± SD), 30.1 ± 17.9 OTUs and 14.8 ± 15.6 OTUs, respectively. Of the 300 phototrophic OTUs observed, 197 were cyanobacteria, 57 were bacillariophytes, 31 were chlorophytes and the other 15 comprised euglenides, eustigmatophyceae, streptophytes, dinoflagellates, a rhodophyta and unknown viridiplantae (see Additional file 2: Table S1). Of the eukaryotic sequences, 47 were eukaryotic 18S sequences, 15 were chloroplast 16S sequences, 28 were mitochondrial 12S and 16S sequences, and 13 remained uncertain (see Additional file 1: Data S1). To reduce variance in alpha-diversity among samples that resulted from differences in sequencing effort (that is, variations in the number of sequencing reads among samples), each sample was rarefied to 40 reads using the rrarefy function in vegan v.2.0 (see Additional file 3: Data S2). We determined that the taxonomic composition within the sub-sampled 40 reads would provide a semi-quantitative measure of relative biomass within each sample.

**Figure 1:**
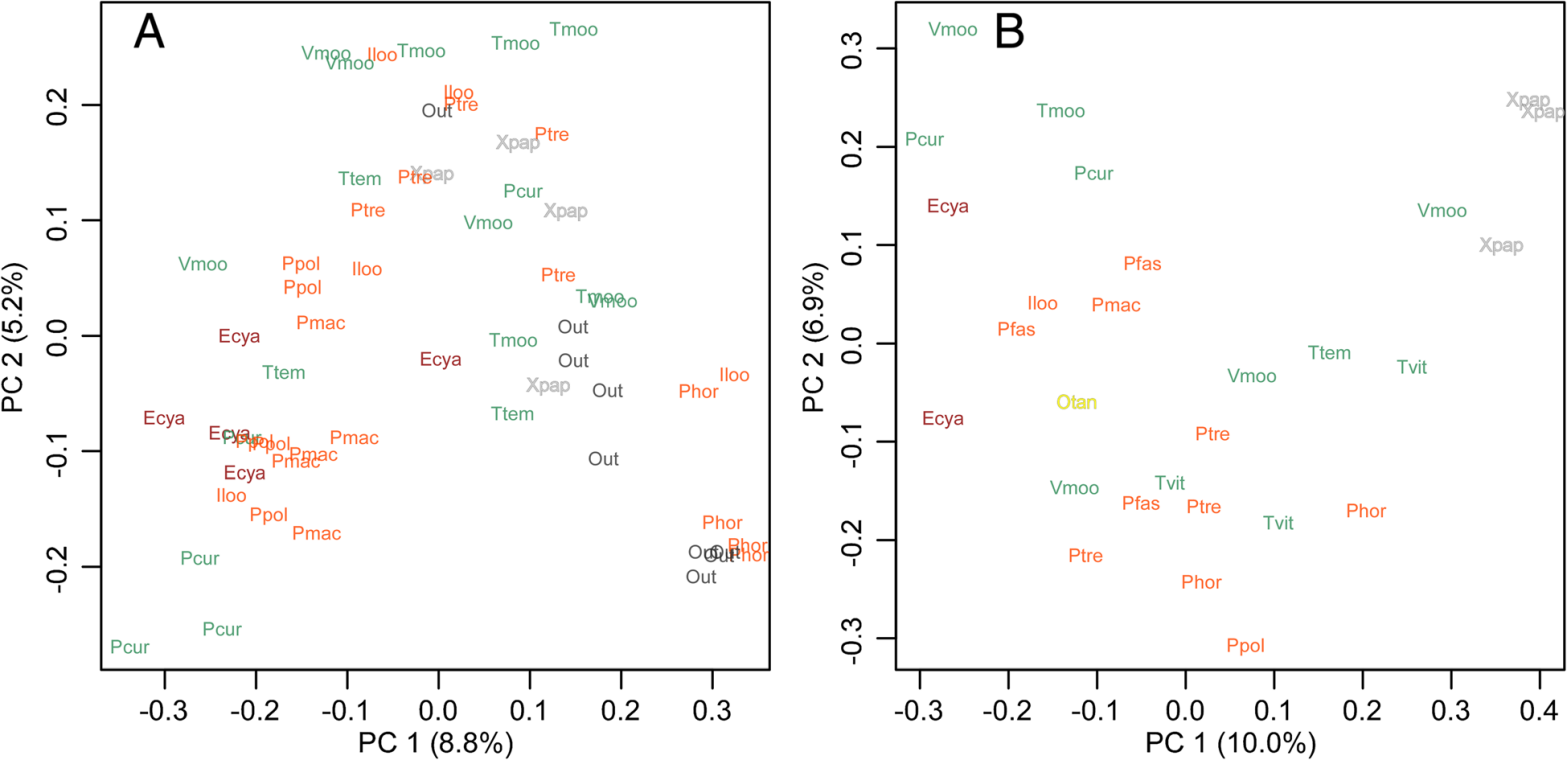
**Principal coordinate analysis among algal farms of herbivorous cichlids (A) and their stomach contents (B).** Axes represent the first two principal coordinates maximising the variance in the data (PC 1 and PC 2). Dissimilarity was calculated using the Canberra distance index. The percentage of the total variance explained by each axis estimated under the broken stick model is shown in parentheses. Abbreviations are listed in Table 1. Orange, green, dark red, grey, and yellow coloured species indicate grazers, browsers, scrapers, scoopers and biters, respectively. Out = periphyton outside the cichlid territories.

Phototroph composition in algal farms varied significantly among cichlid species (Figure 1A; Adonis, *P* <0.05, Additional file 4: Table S2). Phototroph composition in stomach contents also varied significantly among cichlid species (Figure 1B; Adonis, *P* <0.01, Additional file 4: Table S2). These results indicate that variations within species are significantly smaller than variations among species. Among species of the same feeding ecomorph, the phototroph compositions in both their defended farms and stomach contents were widely scattered.

**Figure 2:**
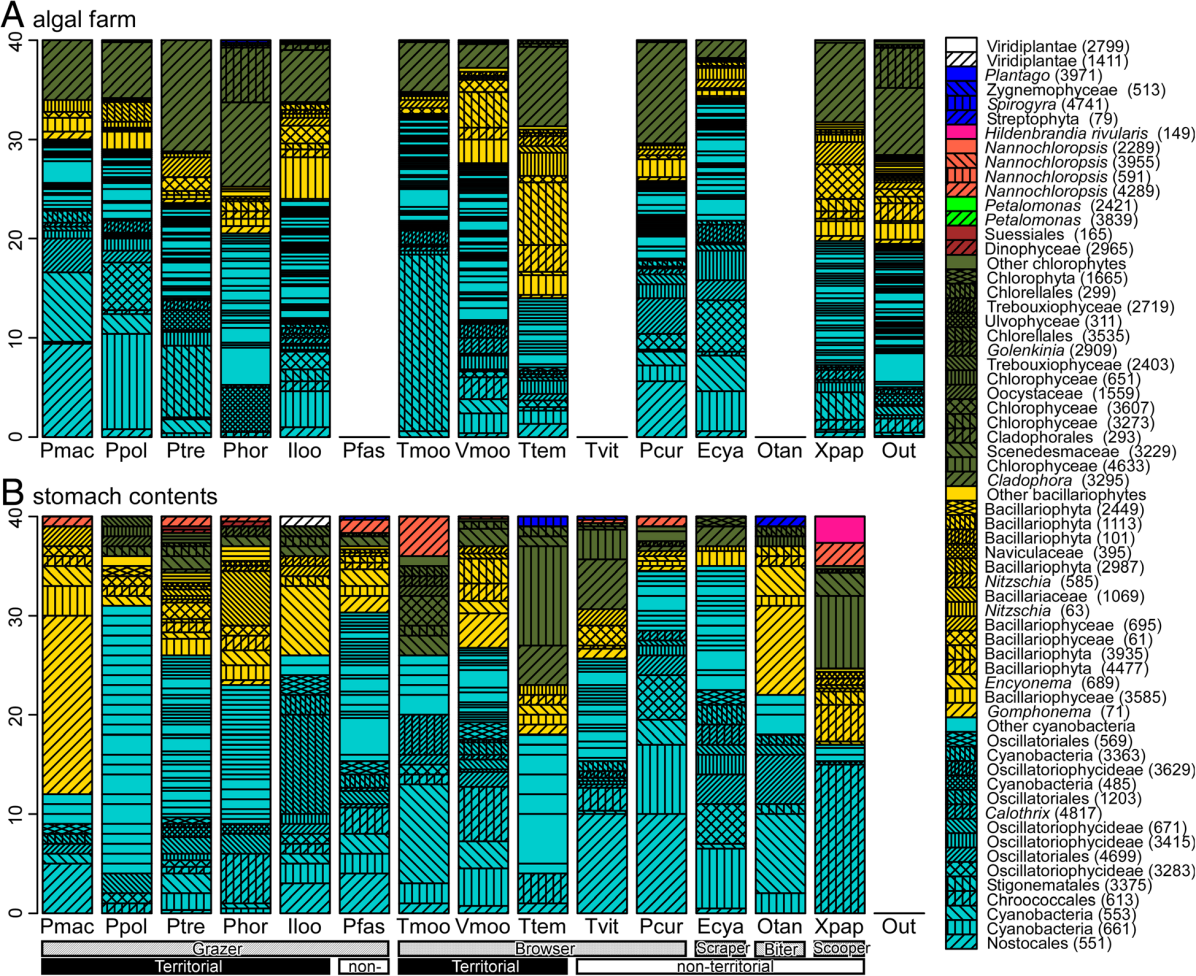
**Phototroph compositions inside and outside the territories of herbivorous cichlids and their stomach contents. A)** Algal farms of herbivorous cichlids and periphyton outside the cichlid territories; **B)** stomach contents of the cichlid fishes. The compositions were averaged over one to five samples. The number of samples is shown in Table 1 and Figure 4.

Phototroph composition in algal farms and stomach contents mostly consisted of three phyla: Chlorophyta, Bacillariophyta and Cyanobacteria (Figure 2). Bacillariophytes were relatively more abundant in the stomachs of grazers compared to both the stomachs of browsers and the algal farms of grazers, indicating that grazers selectively ingested bacillariophytes. The stomach contents of browsers contained more cyanobacteria and chlorophytes than those of grazers. *Cladophora* sp. (OTU #3295) dominated the algal farms of all herbivorous cichlids, but most grazing herbivores seldom ingested this algal species. Only a territorial browser (*T. temporalis*) and non-territorial browser (*T. vittatus*) and a scraper (*E. cyanostictus*) ingested this alga. No cyanobacteria or algae other than the *Cladophora* sp. were dominant throughout the algal farms of herbivorous cichlids. As shown by principal coordinate analysis (PCoA) ordination, the phototroph compositions of algal farms and stomach contents differed considerably among species within the same feeding ecomorph.

**Figure 3:**
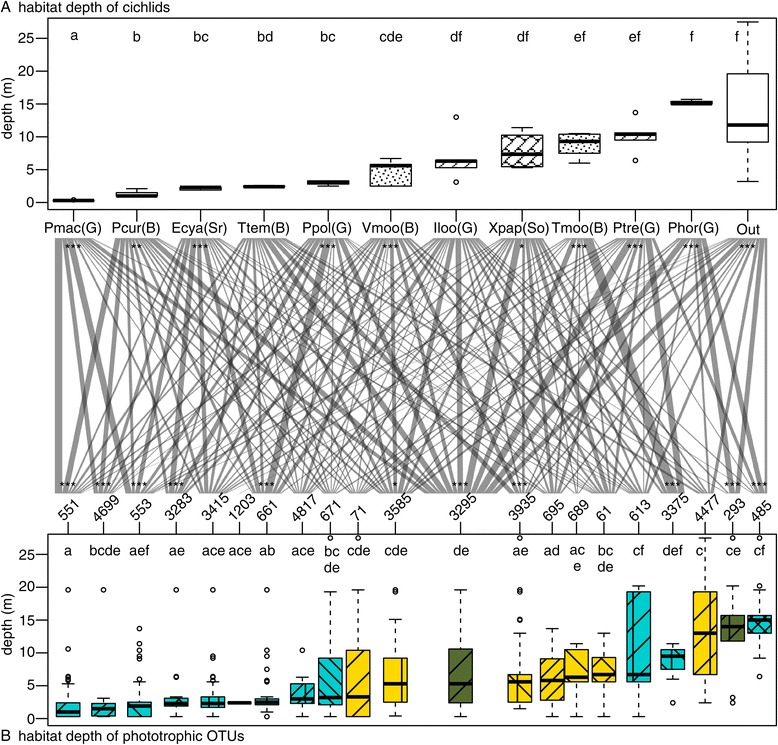
**Habitat depths of cichlids (A) and phototrophic OTUs (B).** Shared letters on box plots indicate no significant differences, and pairs that do not share any letters in common were significantly different by the Tukey *post hoc* test between cichlid species and between OTUs in **(A)** and **(B)**, respectively. Species abbreviations are shown in Table 1. Ecomorphs of cichlids abbreviated in parentheses are as follows: G, grazer; B, browser; Sr, scraper; So, scooper. Colours of OTUs indicate their phyla, and patterns are the same as in Figure 2. Networks between algal farms of cichlid species and OTUs denote the frequency of occurrence with square-root transformation. Asterisks indicate significance in standardised specialisation based on *d’* measures: *=5%, **=1%, ***=0.1%.

The habitat depths of cichlids differed significantly among species (Figure 3, Additional file 5: Table S3), and the depth significantly affected the phototrophic composition (Adonis, *P* <0.001, Additional file 4: Table S2). Two grazers (*P. macrognathus* and *P. polyodon*), two browsers (*P. curvifrons* and *T. temporalis*) and a scraper (*E. cyanostictus*) inhabited the shallowest zone, two grazers (*I. loocki* and *P. trewavasae*), and two browsers (*V. moorii* and *T. moorii*,) and a scooper (*X. papilio*) inhabited the intermediate depth, and a grazer (*P. horii*) occupied the deepest zone (Figure 3A). In addition to the depth segregation between cichlid species, phototrophic OTUs also showed significant variation in habitat depth (Figure 3B, Additional file 6: Table S4, see Additional file 7: Figure S1 for occurrence frequency of the dominant OTUs in various depths). The shallower inhabitants of the orders Nostocales (OTU #551) and Oscillatoriales (#4699), the subclass Oscillatoriophycideae (#3283), and two cyanobacteria (#553 and #661) occurred frequently in algal farms of the cichlids in the sallowest zone. The relatively deep inhabitants, *Cladophora* sp. (#3295), a Stigonematales (#3375), and a diatom (#3935) occurred in the territories of cichlids living in the intermediate depths. A cladophorales (#293) and a cyanobacteria (#485) occurred frequently in the farms of the deepest inhabitant, *P. horii*. Consequently, the standardised specialisation index (*d’*) was significantly higher in the algal farms of cichlid species, with the exceptions of *I. loocki* and *T. temporalis*, than in a random network provided by a null model.

**Figure 4:**
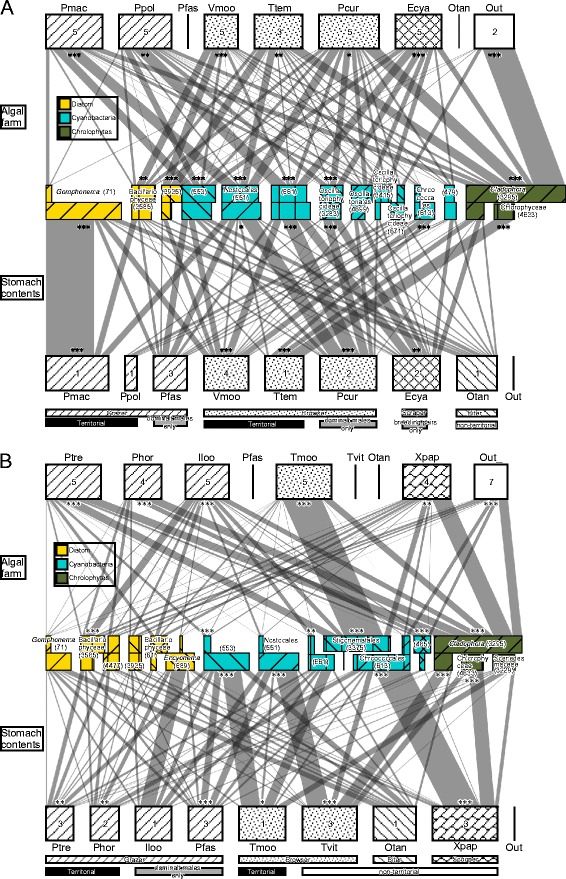
**Networks between herbivorous cichlids and phototrophs in their territories and between phototrophs inside stomachs and fish species. A)** shallow inhabiting cichlids, **B)** deep inhabiting cichlids. Species abbreviations are shown in Table 1. Out = outside the territories. Link width indicates the frequency of occurrence of the OTU in algal farms of cichlids or their stomach contents. Algal and cyanobacteria OTUs that occurred less than 10 times in total are not shown to decrease complexity. Colours of OTUs indicate their phyla, and patterns are the same as in Figure 2. Asterisks indicate significance in standardised specialisation based on *d’* measures: *=5%, **=1%, ***=0.1%. These networks were constructed using the composition averaged over one to five samples, and the numbers of samples are shown in the rectangles.

The bipartite graphs show the occurrence of each phototrophic OTU in the algal farms of herbivorous cichlid species (upper bipartite graphs), and the occurrence of those OTUs in the stomachs of the cichlid species (lower bipartite graphs) living in the shallower area (2.4 ± 1.6 m depth, Figure 4A) and deeper area (11.2 ± 5.4 m depth, Figure 4B). The network structures of the upper and lower graphs were quite different in both shallow and deep inhabitants (similarity matrices were compared between algal farms and stomach contents using the Mantel test, for both shallow and deep inhabitants, *z* =10.72 and 7.52, respectively, both *P* >0.05), which indicates that the cichlid species did not ingest their algal farms randomly, but they utilised phototrophs selectively. Further, for the upper graphs, the network level measure of specialisation *H*_*2*_*’* values (0.20 and 0.27 in shallow and deep inhabitants, respectively) were significantly higher than those calculated for the null models (0.08 ± 0.01 and 0.10 ± 0.01 in shallow and deep inhabitants, respectively; both *P* <0.001), suggesting that a specific structure of phototrophs was established in the algal farms of each cichlid species within the same depth range. For the lower graphs, *H*_*2*_*’* values (0.31 and 0.40 in shallow and deep inhabitants, respectively) were also significantly higher than those calculated for the null models (0.15 ± 0.01 and 0.17 ± 0.02 in shallow and deep inhabitants, respectively; both *P* <0.001), suggesting that the composition of stomach contents varied widely among cichlid species within the same depth range.

**Figure 5:**
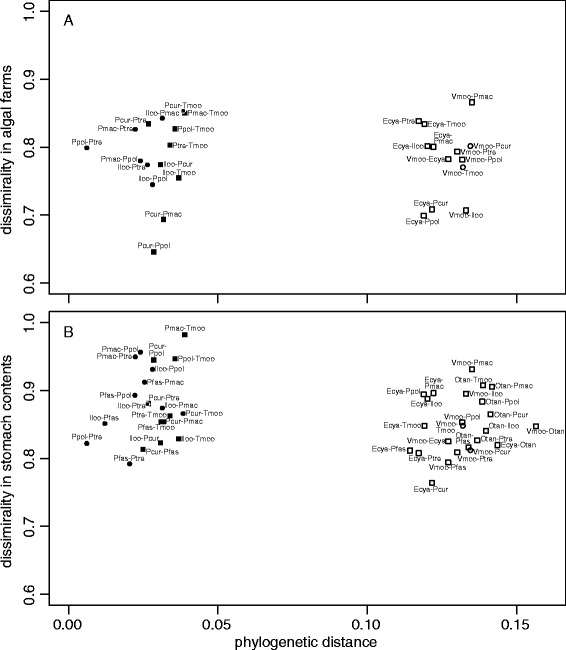
**Pairwise distance-contrast plots between the phylogenetic distance of fishes versus dissimilarity in phototroph compositions. A)** The correlation between the phylogenetic distance of fish species versus dissimilarity in the phototroph compositions of algal farms defended by the herbivorous cichlids, and **B)** pairwise plots between the phylogenetic distance versus dissimilarity in phototroph composition of stomach contents of the herbivorous cichlids. Circles and squares indicate species-pairs of the same feeding ecomorph and of different ecomorphs, respectively. Closed and open symbols indicate species-pairs of the same tribe and of different tribes, respectively. Dissimilarity was calculated using the Canberra distance index. Species abbreviations are listed in Table 1.

**Figure 6:**
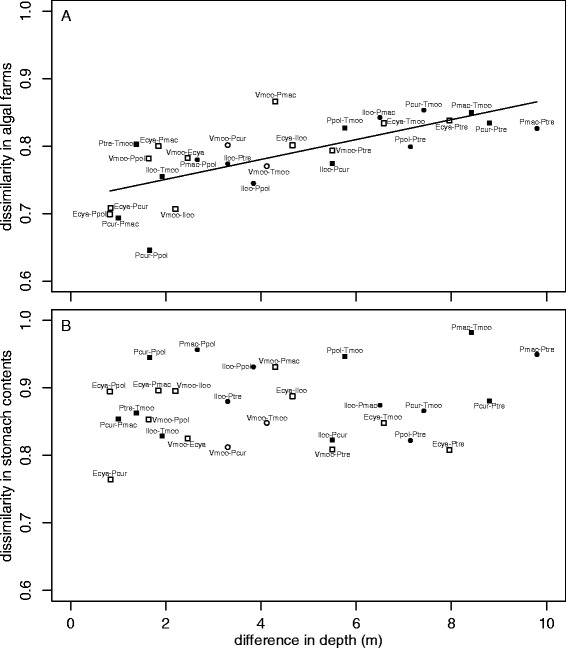
**Pairwise distance-contrast plots between the difference in habitat depth versus dissimilarity in phototroph compositions. A)** The correlation between the difference in habitat depth of fish species versus the dissimilarity in the phototroph compositions of algal farms defended by the herbivorous cichlids, and **B)** pairwise plots between the difference in habitat depth versus the dissimilarity in phototroph composition of stomach contents of the herbivorous cichlids. Circles and squares indicate species-pairs of the same feeding ecomorph and of different ecomorphs, respectively. Closed and open symbols indicate species-pairs of the same tribe and of different tribes, respectively. The solid line is the fitted line for GLM using the difference in depth as a fixed factor. Dissimilarity was calculated using the Canberra distance index. Species abbreviations are listed in Table 1. GLM, generalized linear model.

The lower bipartite graphs in Figure 4A,B show that different phototrophic species or different combinations of phototrophic species contributed to the stomach contents of different cichlid species even within the same depth range. All the cichlid species both living in shallow and deep zones had significant specific links with various phototrophic OTUs. Neither dissimilarity in the algal farm composition or stomach contents in species-pairs was related to the phylogenetic distance of the pairs (Figure 5, Mantel test: *z* = 1.63 for algal farm versus cichlid phylogeny; *z* = 3.19 for stomach contents versus cichlid phylogeny; both *P* >0.05), even between species in the Tropheini tribe, which comprises a single lineage of grazers and a few lineages of browsers (Mantel test: *z* = 0.35 for algal farm; *z* = 0.51 for stomach contents; both *P* >0.05). Dissimilarity in algal farms and stomach contents did not differ between same-tribe pairs and different-tribe pairs, or between same feeding-ecomorph pairs and different ecomorph pairs (generalized linear model (GLM), *P* >0.05 for both factors, Additional file 8: Table S5). On the other hand, dissimilarity in algal farm composition was positively correlated with differences in habitat depth (Figure 6; Mantel test, *z* = 10620.73, *P* <0.001; GLM, *P <*0.01, Additional file 8: Table S5). Dissimilarity in stomach contents of all the cichlids was not related to the difference in their habitat depths.

## Discussion

Phototroph composition in algal farms defended by herbivorous cichlids significantly varied among cichlid species, even among species in the same ecomorphs. This was due in part to habitat depth segregation of cichlids, as the dissimilarity of algal farms increased significantly when the difference in habitat depth increased. Segregation of habitat range by depth among cichlid species has been described in several species pairs of the same ecomorphs [10,23,30,33]. Additionally, the stomach contents of these cichlids were segregated by at least 0.75 in the Canberra dissimilarity index, even between species in the same feeding ecomorph inhabiting the same depth range. The stomach content compositions were quite different from those of the algal farms of the same species. These results indicate that these cichlid species are highly selective when ingesting phototrophs from their specific algal farms, resulting in specific utilisation of phototrophs by each cichlid species. Consequently, similarity in algal farms was relatively high in the same depth range, but the stomach contents differed among species living in the same depth range. Stomach contents of cichlids were so diverse that no significant effect of ecomorph, tribe, habitat depth, and their interactions on the similarities of the stomach contents was detected. These territorial cichlids ingest algae from specific points inside their territories [10,17,48], and, therefore, further study on phototrophic distribution within territories and detailed feeding sites will reveal the mechanism that enables diet segregation among cichlid species of each ecomorph living in the same depth range.

Stomachs of grazers had higher compositions of bacillariophytes, such as *Gomphonema* sp., *Encyonema* sp., and Bacillariophyceae OTU #3585, suggesting that grazers ingest these diatoms selectively. This result is in agreement with previous reports of high proportions of epiphytic unicellular algae in the stomachs of the grazers [10,13,48,49], and with a study that found high activity of a digestive enzyme, laminarinase, which hydrolyses laminarine, the main polysaccharide of diatoms, in the grazer *P. orthognathus* [21]. On the other hand, browsers ingested more cyanobacteria, such as Chroococcales, Oscillatoriales and chlorophytes, such as Scenedesmaceae. This result is in agreement with previous reports of high proportions of filamentous algae and cyanobacteria in the stomachs of browsers based on visual observations of stomach contents [10,13,48,49].

A filamentous green alga, *Cladophora* sp., dominated the farms of most cichlid species, but was not ingested often. *Cladophora* are known to be unpalatable due to their chemical defences and poor amino acid contents [50,51]. Therefore, this alga seems to be utilised as a substratum harbouring epiphytic diatoms for grazers and is a less preferred food item for other herbivorous cichlids.

In Lake Tanganyika, multiple species of herbivorous cichlids coexist sympatrically, but the number of ecomorphs is limited; therefore, the lake has been thought to be a ‘species-saturated’ community where the number of species exceeds the number of available niches [4]. This study, however, shows that these cichlids segregate their habitats on a finer scale, just as multiple *Anolis* lizard species of the same ecomorph specialise in specific thermal microhabitats or specific prey sizes and coexist in the Caribbean islands [52-54].

Our results demonstrate that phylogenetically close cichlid species neither always defend similar or opposite-structured algal farms nor ingest similar or opposite-structured phototrophic compositions. This confirms the hypothesis that niche lability exceeds niche conservatism in a community in which species have a long evolutionary history of ecological interaction, as seen in the adaptive radiation of Caribbean *Anolis* lizards [55]. This also suggests that the flexibility to specialise in microhabitats may drive their divergence and coexistence.

## Conclusions

To date, it has been difficult to classify unicellular and filamentous algae, especially diatoms and cyanobacteria, to the species level, especially in stomach contents, and evidence of dietary segregation in herbivorous cichlids in the same feeding ecomorph is scarce [4,56]. This study revealed that the phototrophic composition of algal farms was quite different among species, even those in the same feeding ecomorph, because of habitat depth segregation among species. In addition, cichlid species selectively fed on phototrophs from their algal farms. As a result, the algal compositions of stomachs differed among species, even those in the same ecomorph inhabiting the same depth range. Therefore, a metagenomic approach revealed food niche separation based on habitat-depth segregation and food preference among coexisting herbivorous cichlids in the same ecomorphs on a rocky shore of Lake Tanganyika.

## Methods

### Sampling

We sampled 16 species of herbivorous cichlids (*Oreochromis tanganicae*, *Xenotilapia papilio*, *Eretmodus cyanostictus*, *Telmatochromis temporalis*, *Telmatochromis vittatus*, *Variabilichromis moorii*, *Interochromis loocki*, *Pseudosimochromis curvifrons*, *Petrochromis famula*, *Petrochromis fasciolatus*, *Petrochromis macrognathus*, *Petrochromis polyodon*, *Petrochromis horii*, *Petrochromis trewavasae*, *Simochromis diagramma*, and *Tropheus moorii*) from Kasenga Point (8°43′ S, 31°08′ E) near Mpulungu, Zambia, on the southern tip of Lake Tanganyika in November 2010, using a gill net (Table 1). Stomach contents were extracted from one to five individuals of each species and immediately preserved in 100% ethanol for molecular analysis. Periphyton samples were simultaneously collected by scuba divers from five territories each of *T. temporalis*, *V. moorii*, *P. macrognathus*, *P. polyodon*, *P. horii*, *P. trewavasae*, *T. moorii*, dominant males of *I. loocki* and *P. curvifrons* (in these species females and inferior males do not have feeding territory, Table 1) and breeding pairs of *E. cyanostictus* and *X. papilio.* Another 13 periphyton samples were collected from outside the cichlid territories using separate sampling bottles to avoid cross-contamination during collection. Each periphyton sample was collected from the territory of different cichlid individuals, and the depth of the sampling location was measured to the nearest 0.1 m using a diving computer. We defined the territory as the area where the territory holder fed on and defended against conspecific and heterospecific herbivores [57]. Whether a site was located within or outside of a cichlid fish territory was determined by 20 minutes of observation immediately prior to sampling. Periphyton samples were immediately preserved in 100% ethanol and stored at −25°C in the laboratory until molecular analysis.

### Metagenomic amplicon sequencing

#### DNA extraction

From each algal farm or stomach content sample, a 2-mm^3^ subsample was pulverised by beating with 4-mm zirconium beads 20 times per second for 2 minutes using a TissueLyser II (Qiagen, Venlo, The Netherlands). Total DNA was extracted from the crushed tissue using the cetyltrimethylammonium bromide (CTAB) method [58].

#### Universal primers, PCR, and pyrosequencing

We sequenced SSU rRNA genes using tag-encoded massively parallel pyrosequencing analysis [59-61]. We aimed to focus on all phototrophs, including cyanobacteria and various algal taxa. The SSU rRNA gene is effective for DNA barcoding of organisms, especially bacteria, because of the massive public database [62]. The primer set, CYA_ALG_F (5′-AAA CTC AAA GRA ATT GAC GG-3′; *Escherichia coli* position 906 to 25) and CYA_ALG_R (5′-GYT ACC TTG TTA CGA CTT C-3′; *E. coli* position 1490 to 1508), was designed to amplify the highly variable V6 region of the 16S rRNA gene [63]*.* The coverage of the primers was evaluated based on *in silico* polymerase chain reaction (PCR) using the Probe Match function in the Ribosomal Database Project (RDP) release 11.2 [64] on 10 June 2014, and the TestPrime 1.0 function [65] within the SILVA release 115 database [62] on 22 May 2014. The coverage of the newly designed primer set was much higher than that of two other primer sets designed for ‘phyto-specific’ 16S rRNA gene amplification [66,67] (see Additional file 9: Figure S2). Although the coverage for eukaryotic algal taxa was limited in the TestPrime results, these coverage values may be underestimated because our focus was on the 16S rRNA gene of plastids, while the eligible sequences in the SILVA database include the 18S rRNA genes of eukaryotes. The primer coverage was determined to be sufficient for demonstrating the differences between the algal/cyanobacterial communities in the algal farms and stomach contents of cichlid species.

For each sample, SSU rRNA gene sequences were amplified using the forward primer CYA_ALG_F fused with the 454 pyrosequencing Adaptor A (5′-CCA TCT CAT CCC TGC GTG TCT CCG ACT CAG-3′) and the 8-mer molecular ID [61], and the reverse primer CYA_ALG_R, with the Ampdirect Plus (Shimadzu Corp., Kyoto, Japan) buffer system and BIOTAQ HS DNA Polymerase (Bioline, London, U.K.). All forward primers were tested using AmplifX_1.5.4, developed by Nicolas Jullien [68] to avoid potential primer-dimers. PCR was conducted using a temperature profile of: 95°C for 10 minutes; 30 cycles at 94°C for 20 seconds, 48°C for 30 seconds, and 72°C for 30 seconds; and a final extension at 72°C for 7 minutes. For each PCR amplicon, DNA concentrations were measured using a Qubit 2.0 Fluorometer (Invitrogen, Carlsbad, CA, USA) and were diluted to 4 ng/μl with Milli-Q water. PCR amplicons were pooled and purified using ExoSAP-IT (GE Healthcare, Little Chalfont, Buckinghamshire, U.K.) and a QIAquick PCR Purification Kit (Qiagen). The SSU rRNA amplicons were subjected to pyrosequencing using a GS Junior sequencer (Roche, Basel, Switzerland). The sequencing was conducted according to the manufacturer’s instructions.

### Assembling of pyrosequencing reads

In total, we obtained 92,559 SSU rRNA gene sequence reads from the pyrosequencing run. We trimmed low-quality 3′ tails, with a minimum quality value of 27. We discarded short reads that were less than 150 base pairs, excluding the forward primer and molecular ID positions. After the elimination of possible chimeras using UCHIME v4.2.40 [69] with a minimum score of 0.1 to report a chimera, a total of 87,898 reads remained [DDBJ: DRA002208]. Reads were assembled using Assams-assembler v0.1.2012.05.24 [70,71], which is a highly parallelised extension of the Minimus assembly pipeline [72]. All reads were assembled with a minimum cut-off similarity of 97% to remove pyrosequencing errors. The resulting 5,073 consensus sequences represented OTUs (see Additional file 1: Data S1). Of the 5,073 consensus reads, 2,663 reads were excluded as singletons because such sequences are putatively erroneous. The rarefaction curves, which were drawn using the R package vegan_2.0 [73], showed that the number of OTUs did not reach saturation for many of the samples (see Additional file 10: Figure S3); however, the rare OTUs may have contributed little as a dietary source for herbivorous fishes.

### Molecular identification of cyanobacteria and algae

Taxonomic assignment of the OTUs was performed by the QCauto method [37], which is known to return the most accurate taxonomic identification results among the existing automated DNA barcoding methods [37], using the software Claident v. 0.1.2012.05.21 [36,37]. The ‘all_genus’ and ‘all_class’ reference sequence databases that were updated on 5 February 2014 and provided by Claident were used as references. The ‘all_genus’ and ‘all_class’ datasets contain all the sequences of the genus or class, respectively, and therefore, ‘all_genus’ was used preferentially, and ‘all_class’ was used complementarily. The benefit of using the QCauto method is that it enables accurate and fully automated taxonomic identification based on BLAST + searches [74] without setting any arbitrary thresholds of sequence identity percentages or E-values [37]. In the taxonomic identification process, the relaxed lowest common ancestor algorithm [60,75] was used. We extracted all phototrophic taxa, as shown in Additional file 1: Data S1. The origins of eukaryotic OTUs were assigned using the Metaxa software to determine whether an OTU originated from nucleic 18S rRNA genes, chloroplast 16S rRNA genes or mitochondrial 12S/16S rRNA genes [76]. The numbers of phototrophic reads for each periphyton and stomach content sample were 159.4 ± 95.1 (average ± SD) and 49.8 ± 76.4, respectively.

To reduce variance in alpha-diversity among samples that resulted from differences in sequencing effort (that is, variations in the number of sequencing reads among samples), we rarefied each sample to 40 reads using the rrarefy function in vegan v.2.0 (see Additional file 3: Data S2). This rarefying method is common for comparisons among samples with various numbers of reads (for example, [77,78]). Although DNA copy number will often differ between species, and primer efficiency will differ among DNA sequences, the number of DNA molecules detected from faeces or digestive tract contents by quantitative PCR can be used as a semi-quantitative measure of prey biomass [79]. Therefore, we determined that the taxonomic composition within the sub-sampled 40 reads would provide a semi-quantitative measure of relative biomass within each sample.

### Network analysis and measure of standardised specialisation

The networks between herbivorous cichlid species and the phototrophs that occurred in their territories, and the networks between cichlid species and phototrophs found inside their stomachs were constructed among species in shallower areas and species in deeper areas respectively, using the R package, bipartite_1.18 [80]. To quantitatively evaluate the association specificity, the *d’* index of the degree of specialisation in the network was calculated [81,82]. The number of reads was summed among samples of each species within each depth zone to form a data matrix for this analysis because here we aimed to determine the species-level interactions between cichlids and phototrophs. Note that intraspecific variation was significantly smaller than interspecific variation, as described below. The observed *d'* index values were compared with those of randomised links in the ‘vaznull’ model [83] with 10,000 permutations. A *d’* index higher than expected by chance indicates association specificity between a cichlid species and phototrophic OTUs, or specificity between a phototrophic OTU and a cichlid species. *H*_*2*_*’* [81], a network-level measure of specialisation based on the deviation of a species’ realised number of interactions from the species’ expected total number of interactions, was also calculated in all bipartite graphs. Observed values were compared with those of the vaznull models with 10,000 permutations. The bipartite package was also used for calculation of indices and making null models [84].

### Statistical analyses

Multivariate analyses of phototroph community structures in cichlid territories were conducted by permutational multivariate analysis of variance using distance matrices calculated with the Canberra index and Bray-Curtis dissimilarities (Adonis; [85]) with the factors of habitat depth and cichlid species. The community structures of stomach contents were also analysed by Adonis, with a factor of cichlid species. The number of null permutations was set to 9,999. PCoA ordination was conducted using the Canberra index and Bray-Curtis dissimilarities among the OTU composition data because PCoA plotting using Canberra distance is recommended for finding clusters in microbial community samples [86]. To compare the habitat depths of each cichlid species, a GLM analysis was conducted with gamma distribution, using the habitat depth as a response variable and cichlid species as an explanatory variable. The Tukey *post hoc* test in R [87] was performed in the multcomp_1.3.3 package (function glht) for the comparisons among cichlid species. To compare the habitat depths of each phototrophic OTU, generalised linear mixed model (GLMM) analysis was conducted with gamma distribution using the depth as a response variable, OTU as a fixed-effect explanatory variable and the cichlid species from whose algal farms the OTU samples were collected as a random-effect explanatory variable. The Tukey *post hoc* test was performed for the comparisons among OTUs. To estimate the extent of divergence and convergence in phototroph utilisation within herbivorous cichlids, we compared the phylogenetic distance between each pair of species to their similarity in phototroph composition of algal farms/stomach contents using the Mantel test with 9,999 permutations. The phylogenetic distance was calculated by the cophenetic () function in the ape package for R based on the mitochondrial ND2 and nuclear *ednrb1* and *phpt1* sequences [4]. The Canberra distance in phototroph composition of algal farms and those of stomach contents were compared using the Mantel test with 9,999 permutations, and the dissimilarities of algal farms and stomach contents were compared with differences in habitat depth using the Mantel test with 9,999 permutations. To test the effect of the differences in tribe, ecomorph and habitat depth on the similarity of algal farm and stomach contents composition, a GLM analysis was conducted with gamma distribution, using the Canberra distance index as a response variable and the differences in tribe, ecomorph and habitat depth as explanatory variables. All analyses were conducted using R_3.1.0 [88]. The two indices of dissimilarity resulted in similar outcomes in all analyses; therefore, the results using Canberra are shown hereafter, and those using Bray-Curtis are provided in Additional file 11: Figure S4, Additional file 12: Figure S5, Additional file 13: Figure S6.

## Additional files

Additional file 1: Data S1. Summary of pyrosequenced SSU rRNA gene sequence reads that passed quality filtering. OTUs observed in the algal farms and stomachs of territorial herbivorous cichlid fishes in Lake Tanganyika. Additional file 2: Table S1. Summary of phototrophic OTUs detected in our pyrosequencing analysis. Additional file 3: Data S2. Table of rarefied phototrophic OTUs used for analyses. Additional file 4: Table S2. Result of Adonis on distance matrices of algal composition in algal farms defended by territorial cichlids, and the stomach contents of herbivorous cichlids. Distances were calculated by Canberra distance index and Bray-Curtis dissimilarity index. DF, degree of freedom; NS, not significant. Additional file 5: Table S3. Summary of GLM testing for the effect of cichlid species on their habitat depth. Additional file 6: Table S4. Summary of GLMM testing for the effects of phototrophic OTUs on their habitat depths. Cichlid individual, from whose territory each OTU was collected, was included as a random effect. Additional file 7: Figure S1. Occurrence frequencies of the dominant phototrophic OTUs in various depths. Additional file 8: Table S5. Summary of GLMs testing for the effect of difference in tribe, ecomorph, and habitat depth on the algal farm and stomach contents similarity. Ecomorph combination (pairs of the same ecomorph or those of different ecomorphs) and tribe combination (pairs of the same tribe or those of different tribes) are included as two fixed effects. A, Canberra index; B, Bray-Curtis dissimilarity. NS, not significant. Additional file 9: Figure S2. Coverage of primer pairs for algal and cyanobacteria SSU rDNA. Forward and reverse primers: CYA_ALG_F and CYA_ALG_R (A, B); PSf (5′-GGG ATT AGA TAC CCC WGT AGT CCT-3′) and Ur (5′-ACG GYT ACC TTG TTA CGA CTT-3′) from [66] (C, D); and PSf and a universal bacteria primer (5′-TAC GGY TAC CTT GTT ACG ACT T-3′) from [67] (E, F). The percentages of sequences amplified by Probe Match of RDP Release 11.2 are shown in A, C and E*,* and those amplified by TestPrime 1.0 of SILVA are shown in B, D and F. Zero, one, or two nucleotide mismatches between the target primer and database sequences were allowed in each analysis. Numbers in parentheses indicate the numbers of eligible sequences that attempted to match with the primer pair. Additional file 10: Figure S3. Rarefaction curves of OTUs based on the number of reads. **A)** algal farms of cichlid fishes, **B)** stomach contents and **C)** rarefaction curve of OTUs based on the number of samples for algal farms of cichlid fishes (blue curve) and stomach contents (red curve). Species abbreviations are listed in Table 1. The shaded area represents the standard deviation obtained from 100 shuffles of sample-ID order. Additional file 11: Figure S4. Principal coordinate analysis among algal farms of herbivorous cichlids **(A)** and their stomach contents **(B)**. Dissimilarity was calculated using the Bray-Curtis dissimilarity index. Axes represent the first two principal coordinates maximising the variance in the data (PC 1 and PC 2). The percentage of the total variance explained by each axis estimated under the broken stick model is shown in parentheses. Species abbreviations are listed in Table 1. Orange-, green-, dark red-, grey-, and yellow-coloured species indicate grazers, browsers, scrapers, scoopers, and biters, respectively. Out = periphyton outside the cichlid territories. Additional file 12: Figure S5. Pairwise distance-contrast plots between phylogenetic distance of fishes versus dissimilarity in phototroph compositions. **A)** The correlation between the phylogenetic distance of fish species versus dissimilarity in the phototroph compositions of algal farms defended by the herbivorous cichlids, and **B)** pairwise plots between the phylogenetic distance versus dissimilarity in phototroph composition of stomach contents of the herbivorous cichlids. Circles and squares indicate species-pairs of the same feeding ecomorph and of different ecomorphs, respectively. Closed and open symbols indicate species-pairs of the same tribe and of different tribes, respectively. Dissimilarity was calculated using the Bray-Curtis dissimilarity index. Species abbreviations are listed in Table 1. Additional file 13: Figure S6. Pairwise distance-contrast plots between difference in habitat depth versus dissimilarity in phototroph compositions. **A)** The correlation between the difference in habitat depth of fish species versus the dissimilarity in the phototroph compositions of algal farms defended by the herbivorous cichlids, and **B)** pairwise plots between the difference in habitat depth versus the dissimilarity in phototroph composition of stomach contents of the herbivorous cichlids. Circles and squares indicate species-pairs of the same feeding ecomorph and of different ecomorphs, respectively. Closed and open symbols indicate species-pairs of the same tribe and of different tribes, respectively. Dissimilarity was calculated using the Bray-Curtis dissimilarity index. Species abbreviations are listed in Table 1.
