# Supplementary material for: Diet disparity among sympatric herbivorous cichlids in the same ecomorphs in Lake Tanganyika: amplicon pyrosequences on algal farms and stomach contents
Source: BMC Biol. 2014 Oct 29;12:90. doi: 10.1186/s12915-014-0090-4 (PMC4228161; doi:10.1186/s12915-014-0090-4)
Supplement: Additional file 2: Table S1. — Summary of phototrophic OTUs detected in our pyrosequencing analysis. [file 12915_2014_90_MOESM2_ESM.pdf]

Table S1. Summary of phototrophic OTUs

taxa	number of OTUs
Cyanobacteria	197
Bacillariophyta	57
Chlorophyta	31
Eustigmatophyceae	4
Streptophyta	4
Euglenida	2
Dinoflagellata	2
unknown viridiplantae	2
Rhodophyta	1
total	300
