# Supplementary material for: Diet disparity among sympatric herbivorous cichlids in the same ecomorphs in Lake Tanganyika: amplicon pyrosequences on algal farms and stomach contents
Source: BMC Biol. 2014 Oct 29;12:90. doi: 10.1186/s12915-014-0090-4 (PMC4228161; doi:10.1186/s12915-014-0090-4)
Supplement: Additional file 4: Table S2. — Result of Adonis on distance matrices of algal composition in algal farms defended by territorial cichlids, and the stomach contents of herbivorous cichlids. Distances were calculated by Canberra distance index and Bray-Curtis dissimilarity index. DF, degree of freedom; NS, not significant. [file 12915_2014_90_MOESM4_ESM.pdf]

Table S2. Result of Adonis on distance matrices of algal composition in algal farms defended by territorial cichlids, and the stomach contents of herbivorous cichlids. Distances were calculated by Canberra distance and Bray-Curtis dissimilarity index. DF, degree of freedom; NS, not significant.

	DF	Sums of squares	Mean squares	*F* Model	R^2	*p*
A. Canberra						
Algal farm						
Depth	1	0.012	0.012	10.307	0.149	< 0.001
Species	10	0.020	0.002	1.702	0.246	< 0.05
Depth × Species	10	0.015	0.002	1.287	0.186	NS
Residuals	29	0.034	0.001		0.419	
Total	50	0.081			1.000	
Stomach contents						
Canberra						
Species	13	5.795	0.446	1.138	0.514	< 0.01
Residuals	14	5.485	0.392		0.486	
Total	27	11.280			1.000	
B. Bray-Curtis						
Algal farm						
Depth	1	0.049	0.049	9.969	0.152	< 0.001
Species	10	0.078	0.008	1.570	0.239	< 0.05
Depth × Species	10	0.055	0.005	1.105	0.168	NS
Residuals	29	0.143	0.005		0.441	
Total	50	0.325			1.000	
Stomach contents						
Species	13	5.374	0.413	1.397	0.565	< 0.01
Residuals	14	4.141	0.296		0.435	
Total	27	9.515			1.000	
