# Supplementary material for: Diet disparity among sympatric herbivorous cichlids in the same ecomorphs in Lake Tanganyika: amplicon pyrosequences on algal farms and stomach contents
Source: BMC Biol. 2014 Oct 29;12:90. doi: 10.1186/s12915-014-0090-4 (PMC4228161; doi:10.1186/s12915-014-0090-4)
Supplement: Additional file 5: Table S3. — Summary of GLM testing for the effect of cichlid species on their habitat depth. [file 12915_2014_90_MOESM5_ESM.pdf]

Table S3. Summary of GLM testing for the effect of cichlid species on their habitat depth.

	Estimate	Std. Error	*t* value	*p*
(Intercept)	3.125	0.516	6.061	< 0.001
Pcur	-2.367	0.531	-4.462	< 0.001
Ecya	-2.662	0.521	-5.107	< 0.001
Ttem	-2.720	0.523	-5.202	< 0.001
Ppol	-2.789	0.519	-5.379	< 0.001
Vmoo	-2.909	0.517	-5.628	< 0.001
Iloo	-2.978	0.516	-5.770	< 0.001
Xpap	-2.998	0.516	-5.808	< 0.001
Tmoo	-3.011	0.516	-5.835	< 0.001
Ptre	-3.026	0.516	-5.866	< 0.001
Phor	-3.059	0.516	-5.932	< 0.001
Out	-3.052	0.516	-5.918	< 0.001
