# Supplementary material for: Diet disparity among sympatric herbivorous cichlids in the same ecomorphs in Lake Tanganyika: amplicon pyrosequences on algal farms and stomach contents
Source: BMC Biol. 2014 Oct 29;12:90. doi: 10.1186/s12915-014-0090-4 (PMC4228161; doi:10.1186/s12915-014-0090-4)
Supplement: Additional file 6: Table S4. — Summary of GLMM testing for the effects of phototrophic OTUs on their habitat depths. Cichlid individual, from whose territory each OTU was collected, was included as a random effect. [file 12915_2014_90_MOESM6_ESM.pdf]

Table S4. Summary of GLMM testing for the effects of phototrophic OTUs on their habitat depths. Cichlid individual from whose territory each OTU was collected was included as a random effect.

	Estimate	Std. Error	*t* value	*p*
(Intercept)	0.602	0.280	2.146	< 0.05
OTU #4699	-0.092	0.024	-3.812	< 0.001
OTU #553	-0.060	0.019	-3.090	< 0.01
OTU #3283	-0.044	0.021	-2.047	< 0.05
OTU #3415	-0.055	0.022	-2.534	< 0.05
OTU #1203	0.213	0.164	1.297	NS
OTU #661	-0.023	0.021	-1.114	NS
OTU #4817	-0.054	0.022	-2.505	< 0.05
OTU #671	-0.071	0.020	-3.469	< 0.001
OTU #71	-0.093	0.019	-4.880	< 0.001
OTU #3585	-0.082	0.017	-4.729	< 0.001
OTU #3295	-0.073	0.017	-4.321	< 0.001
OTU #3935	-0.060	0.018	-3.328	< 0.001
OTU #695	-0.048	0.019	-2.459	< 0.05
OTU #689	-0.078	0.027	-2.844	< 0.01
OTU #61	-0.071	0.018	-3.917	< 0.001
OTU #613	-0.098	0.018	-5.533	< 0.001
OTU #3375	-0.077	0.017	-4.435	< 0.001
OTU #4477	-0.101	0.017	-5.867	< 0.001
OTU #293	-0.088	0.017	-5.064	< 0.001
OTU #485	-0.096	0.017	-5.574	< 0.001
