# Supplementary figures and images for: Diet disparity among sympatric herbivorous cichlids in the same ecomorphs in Lake Tanganyika: amplicon pyrosequences on algal farms and stomach contents
Source: BMC Biol. 2014 Oct 29;12:90. doi: 10.1186/s12915-014-0090-4 (PMC4228161; doi:10.1186/s12915-014-0090-4)

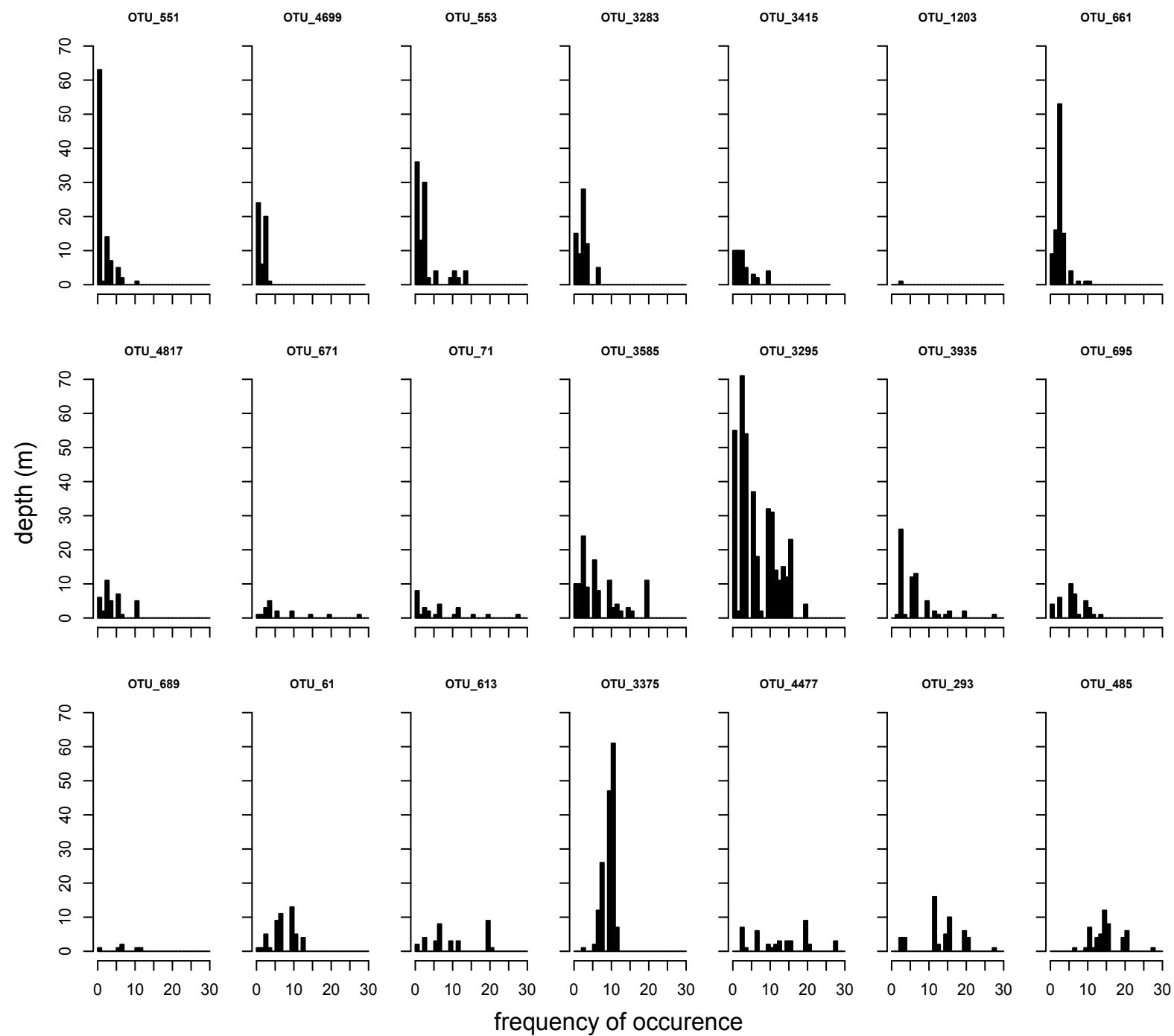

Figure S1

Supplement: Additional file 7: Figure S1. — Occurrence frequencies of the dominant phototrophic OTUs in various depths. [file 12915_2014_90_MOESM7_ESM.pdf]

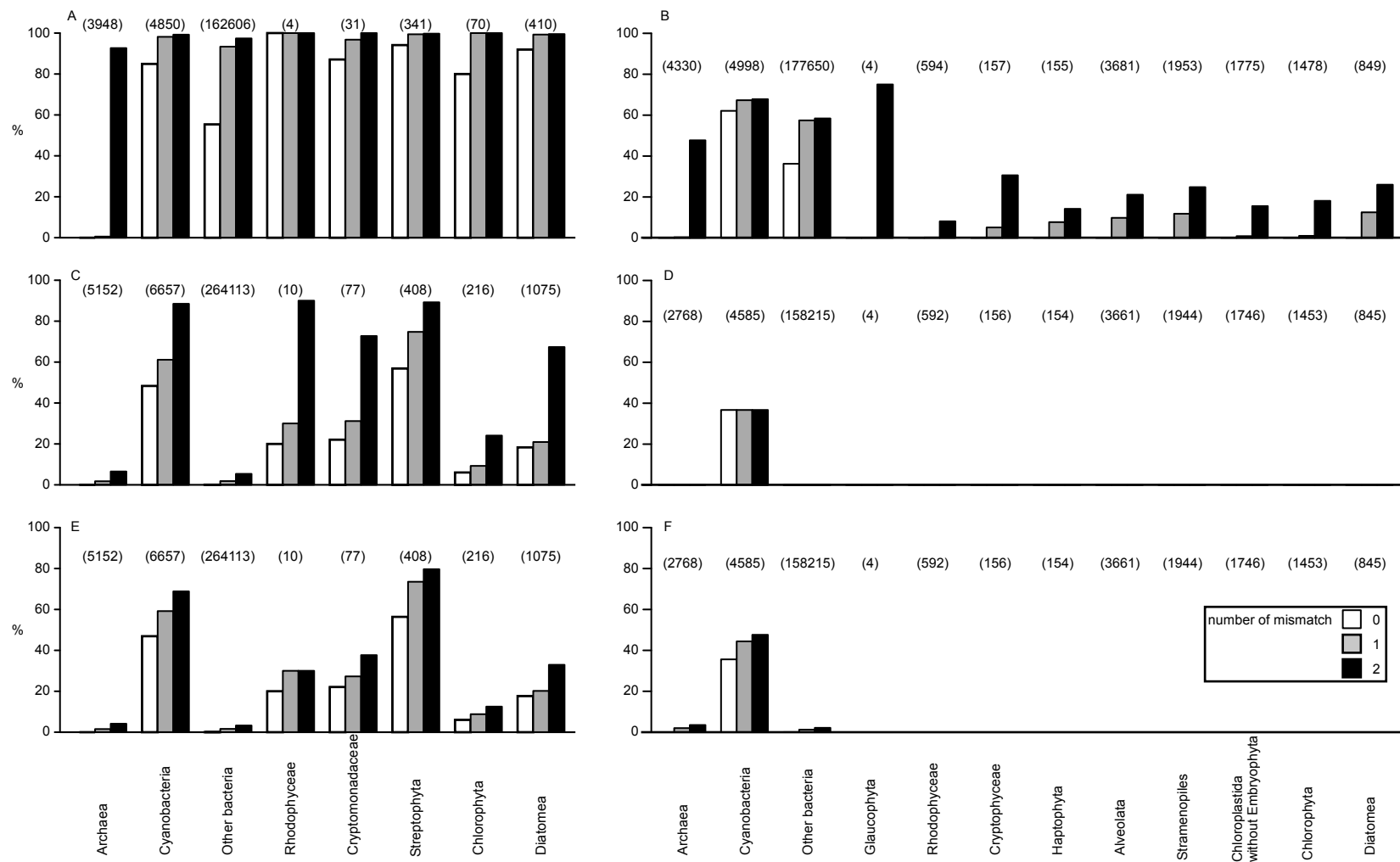

Figure S2

Supplement: Additional file 9: Figure S2. — Coverage of primer pairs for algal and cyanobacteria SSU rDNA. Forward and reverse primers: CYA_ALG_F and CYA_ALG_R (A, B); PSf (5′-GGG ATT AGA TAC CCC WGT AGT CCT-3′) and Ur (5′-ACG GYT ACC TTG TTA CGA CTT-3′) from [66] (C, D); and PSf and a universal bacteria primer (5′-TAC GGY TAC CTT GTT ACG ACT T-3′) from [67] (E, F). The percentages of sequences amplified by Probe Match of RDP Release 11.2 are shown in A, C and E, and those amplified by TestPrime 1.0 of SILVA are shown in B, D and F. Zero, one, or two nucleotide mismatches between the target primer and database sequences were allowed in each analysis. Numbers in parentheses indicate the numbers of eligible sequences that attempted to match with the primer pair. [file 12915_2014_90_MOESM9_ESM.pdf]

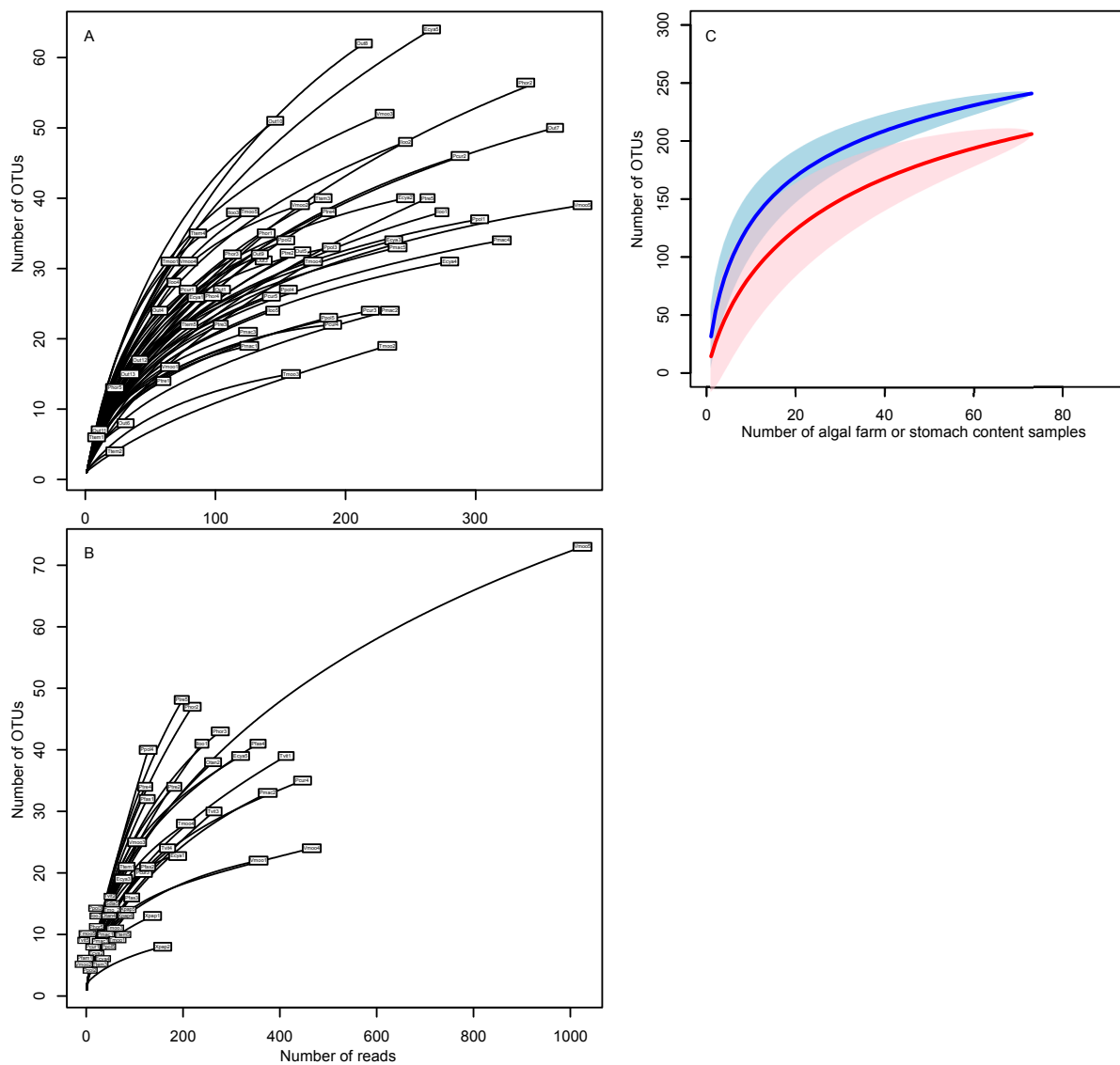

Figure S3

Supplement: Additional file 10: Figure S3. — Rarefaction curves of OTUs based on the number of reads. A) algal farms of cichlid fishes, B) stomach contents and C) rarefaction curve of OTUs based on the number of samples for algal farms of cichlid fishes (blue curve) and stomach contents (red curve). Species abbreviations are listed in Table 1. The shaded area represents the standard deviation obtained from 100 shuffles of sample-ID order. [file 12915_2014_90_MOESM10_ESM.pdf]

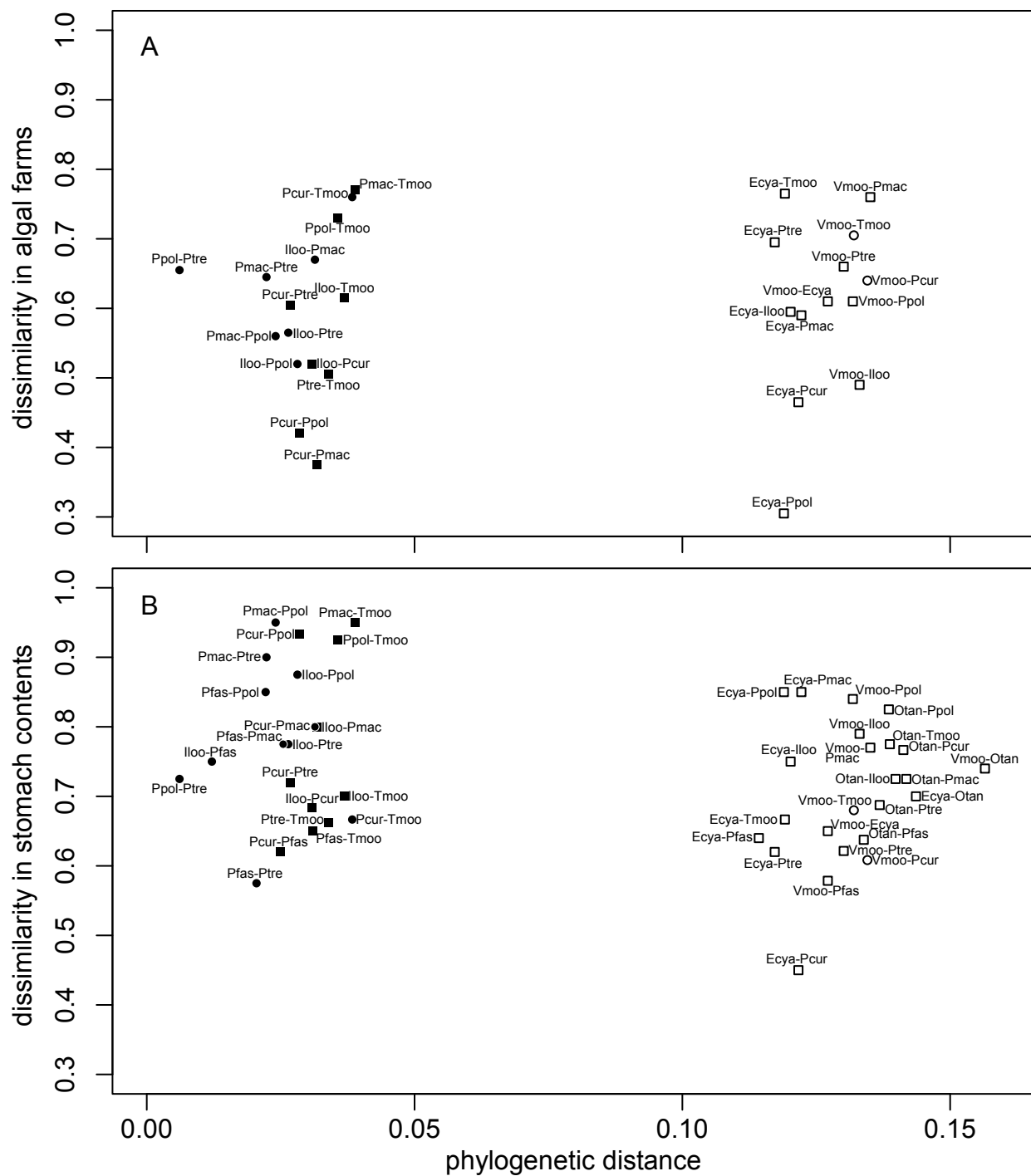

Figure S5

Supplement: Additional file 12: Figure S5. — Pairwise distance-contrast plots between phylogenetic distance of fishes versus dissimilarity in phototroph compositions. A) The correlation between the phylogenetic distance of fish species versus dissimilarity in the phototroph compositions of algal farms defended by the herbivorous cichlids, and B) pairwise plots between the phylogenetic distance versus dissimilarity in phototroph composition of stomach contents of the herbivorous cichlids. Circles and squares indicate species-pairs of the same feeding ecomorph and of different ecomorphs, respectively. Closed and open symbols indicate species-pairs of the same tribe and of different tribes, respectively. Dissimilarity was calculated using the Bray-Curtis dissimilarity index. Species abbreviations are listed in Table 1. [file 12915_2014_90_MOESM12_ESM.pdf]
