# Supplementary material for: Diet disparity among sympatric herbivorous cichlids in the same ecomorphs in Lake Tanganyika: amplicon pyrosequences on algal farms and stomach contents
Source: BMC Biol. 2014 Oct 29;12:90. doi: 10.1186/s12915-014-0090-4 (PMC4228161; doi:10.1186/s12915-014-0090-4)
Supplement: Additional file 8: Table S5. — Summary of GLMs testing for the effect of difference in tribe, ecomorph, and habitat depth on the algal farm and stomach contents similarity. Ecomorph combination (pairs of the same ecomorph or those of different ecomorphs) and tribe combination (pairs of the same tribe or those of different tribes) are included as two fixed effects. A, Canberra index; B, Bray-Curtis dissimilarity. NS, not significant. [file 12915_2014_90_MOESM8_ESM.pdf]

Table S5. Summary of GLMs testing for the effect of difference in tribe, ecomorph, and habitat depth on algal farm and stomach contents similarity. Ecomorph combination (pairs of the same ecomorph or those of different ecomorphs) and tribe combination (pairs of the same tribe or those of different tribes) are included as two fixed effects. A, Canberra index; B, Bray-Curtis dissimilarity. NS, not significant.

	Estimate	Std. Error	*t* value	*p*
A. Canberra index				
Algal farm				
(Intercept)	0.722	0.023	31.678	< 0.001
Same tribe pair	-0.022	0.034	-0.639	NS
Difference in depth	0.017	0.005	3.182	< 0.01
Same ecomorph pair	0.205	0.267	0.768	NS
Same tribe × difference in depth	-0.000	0.007	-0.049	NS
Same tribe × same ecomorph	-0.168	0.272	-0.617	NS
Same ecomorph × difference in depth	-0.055	0.072	-0.773	NS
Same tribe × same ecomorph × difference in depth	0.050	0.072	0.689	NS
Stomach contents				
(Intercept)	0.866	0.030	28.713	< 0.001
Same tribe pair	-0.008	0.045	-0.170	NS
Difference in depth	-0.003	0.007	-0.418	NS
Same ecomorph pair	-0.199	0.354	-0.561	NS
Same tribe × difference in depth	0.010	0.010	1.082	NS
Same tribe × same ecomorph	0.261	0.360	0.726	NS
Same ecomorph × difference in depth	0.047	0.095	0.494	NS
Same tribe × same ecomorph × difference in depth	-0.058	0.095	-0.612	NS
B. Bray-Curtis similarity				
Algal farm				
(Intercept)	0.447	0.050	8.968	< 0.001
Same tribe pair	-0.014	0.075	-0.191	NS
Difference in depth	0.042	0.012	3.543	< 0.01
Same ecomorph pair	-0.069	0.585	-0.118	NS
Same tribe × difference in depth	-0.011	0.016	-0.670	NS
Same tribe × same ecomorph	0.129	0.594	0.216	NS
Same ecomorph × difference in depth	0.037	0.157	0.239	NS
Same tribe × same ecomorph × difference in depth	-0.046	0.158	-0.291	NS
Stomach contents				
(Intercept)	0.764	0.068	11.312	< 0.001
Same tribe pair	-0.002	0.101	-0.016	NS
Difference in depth	-0.014	0.016	-0.872	NS
Same ecomorph pair	-0.444	0.792	-0.560	NS
Same tribe × difference in depth	0.022	0.022	1.024	NS
Same tribe × same ecomorph	0.562	0.805	0.698	NS
Same ecomorph × difference in depth	0.101	0.212	0.478	NS
Same tribe × same ecomorph × difference in depth	-0.121	0.213	-0.567	NS
