# Supplementary material for: Diet disparity among sympatric herbivorous cichlids in the same ecomorphs in Lake Tanganyika: amplicon pyrosequences on algal farms and stomach contents
Source: BMC Biol. 2014 Oct 29;12:90. doi: 10.1186/s12915-014-0090-4 (PMC4228161; doi:10.1186/s12915-014-0090-4)
Supplement: Additional file 11: Figure S4. — Principal coordinate analysis among algal farms of herbivorous cichlids (A) and their stomach contents (B). Dissimilarity was calculated using the Bray-Curtis dissimilarity index. Axes represent the first two principal coordinates maximising the variance in the data (PC 1 and PC 2). The percentage of the total variance explained by each axis estimated under the broken stick model is shown in parentheses. Species abbreviations are listed in Table 1. Orange-, green-, dark red-, grey-, and yellow-coloured species indicate grazers, browsers, scrapers, scoopers, and biters, respectively. Out = periphyton outside the cichlid territories. [file 12915_2014_90_MOESM11_ESM.pdf]

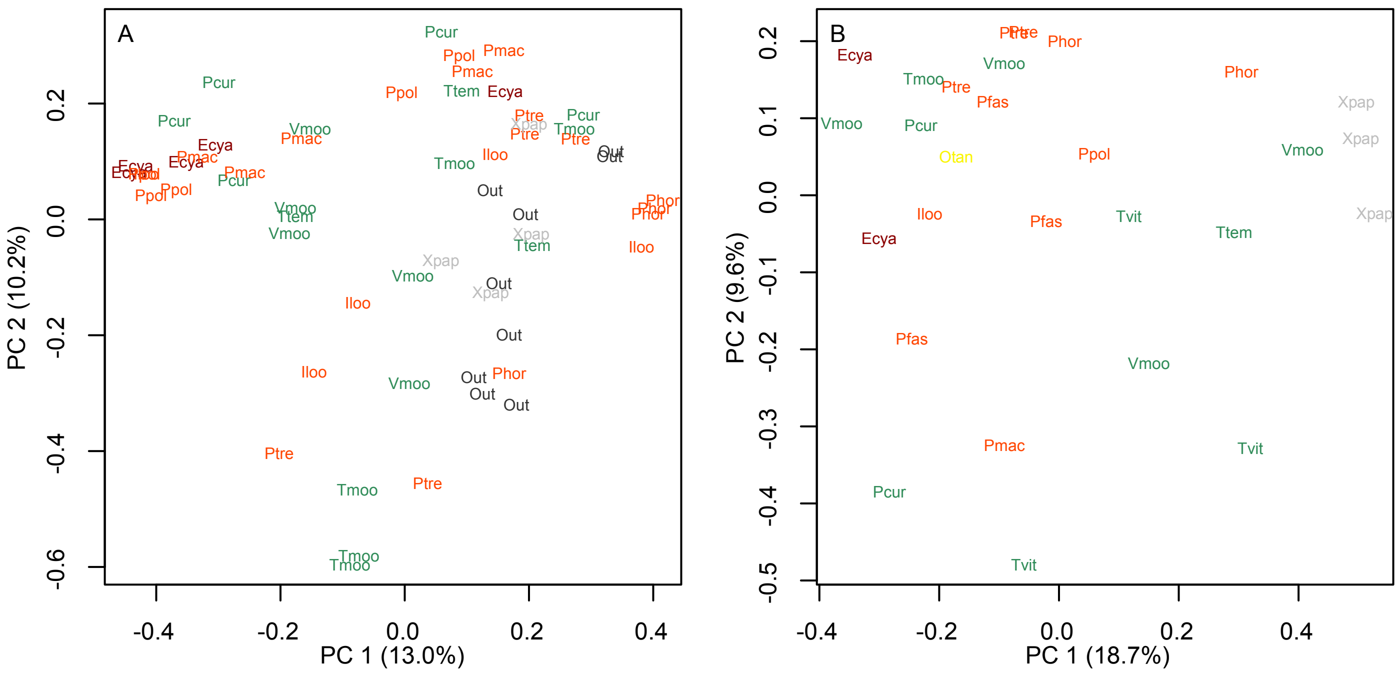
A
B
PC 1 (13.0%)
PC 2 (10.2%)
PC 1 (18.7%)
PC 2 (9.6%)
-0.4
-0.2
0.0
0.2
0.4
-0.6
0.1
-0.1
-0.3
-0.5
Pcur
Ppol
Pmac
Ttem
Ecya
Ptre
Xpap
Tmoo
Out
Vmoo
Iloo
Phor
Pfas
Otan
Tvit

Figure S4
