# Supplementary material for: Diet disparity among sympatric herbivorous cichlids in the same ecomorphs in Lake Tanganyika: amplicon pyrosequences on algal farms and stomach contents
Source: BMC Biol. 2014 Oct 29;12:90. doi: 10.1186/s12915-014-0090-4 (PMC4228161; doi:10.1186/s12915-014-0090-4)
Supplement: Additional file 13: Figure S6. — Pairwise distance-contrast plots between difference in habitat depth versus dissimilarity in phototroph compositions. A) The correlation between the difference in habitat depth of fish species versus the dissimilarity in the phototroph compositions of algal farms defended by the herbivorous cichlids, and B) pairwise plots between the difference in habitat depth versus the dissimilarity in phototroph composition of stomach contents of the herbivorous cichlids. Circles and squares indicate species-pairs of the same feeding ecomorph and of different ecomorphs, respectively. Closed and open symbols indicate species-pairs of the same tribe and of different tribes, respectively. Dissimilarity was calculated using the Bray-Curtis dissimilarity index. Species abbreviations are listed in Table 1. [file 12915_2014_90_MOESM13_ESM.pdf]

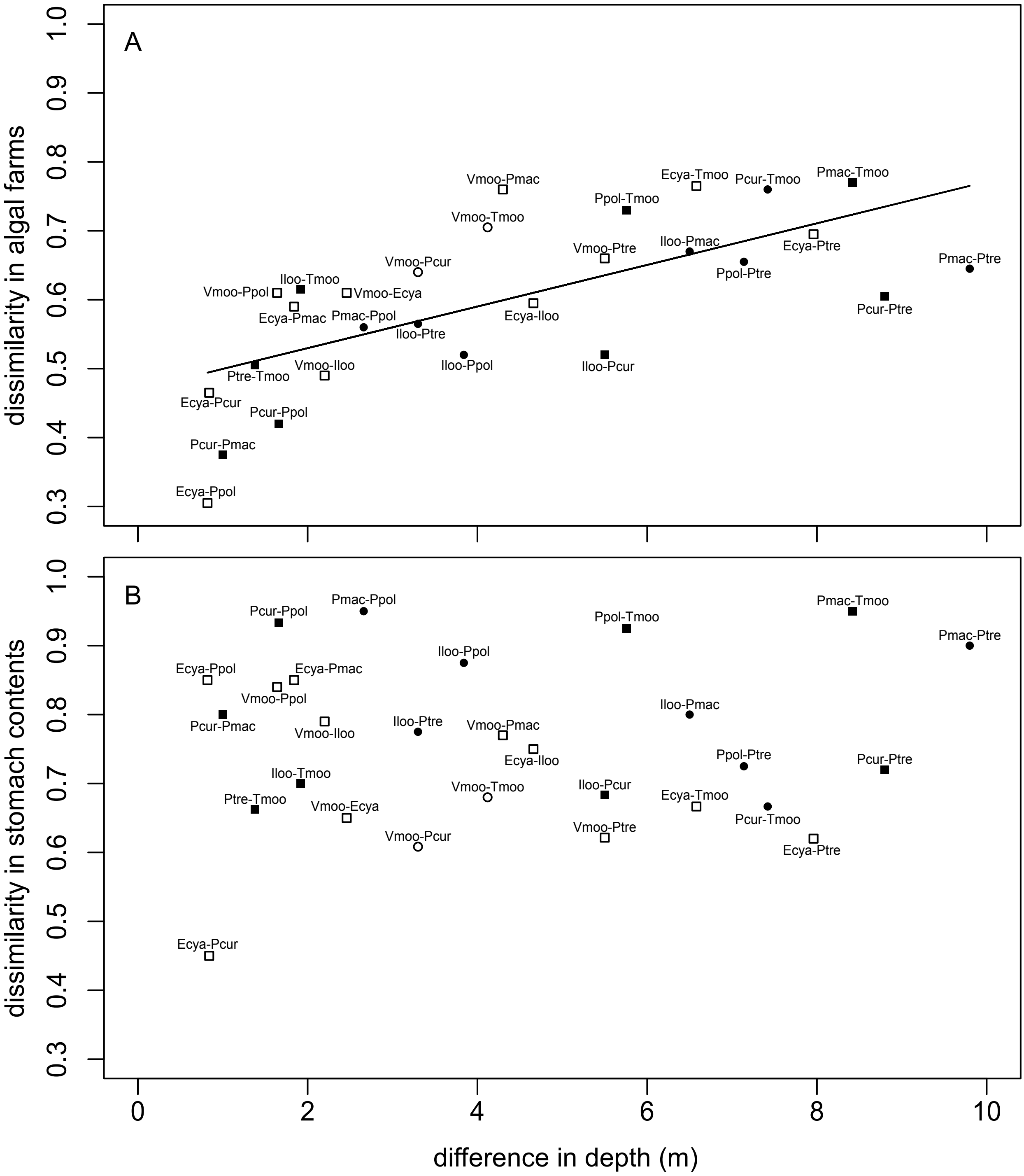

A
B
dissimilarity in algal farms
dissimilarity in stomach contents
difference in depth (m)
0.3
0.4
0.5
0.6
0.7
0.8
0.9
1.0
0
2
4
6
8
10
Vmoo-Pmac
Ecya-Tmoo
Pcur-Tmoo
Pmac-Tmoo
Ppol-Tmoo
Vmoo-Tmoo
Vmoo-Ptre
Iloo-Pmac
Ecya-Ptre
Pmac-Ptre
Vmoo-Pcur
Ppol-Ptre
Iloo-Tmoo
Vmoo-Ppol
Vmoo-Ecya
Ecya-Pmac
Pmac-Ppol
Ecya-Iloo
Iloo-Ptre
Pcur-Ptre
Iloo-Ppol
Iloo-Pcur
Ptre-Tmoo
Vmoo-Iloo
Ecya-Pcur
Pcur-Ppol
Pcur-Pmac
Ecya-Ppol


Figure S6
